# The ultimate exercise countermeasure for long‐duration spaceflight?

**DOI:** 10.1113/EP092820

**Published:** 2025-05-27

**Authors:** Donald E. Watenpaugh, Alan R. Hargens

**Affiliations:** ^1^ Department of Physiology and Anatomy University of North Texas Health Science Center Fort Worth Texas USA; ^2^ Department of Bioengineering University of Texas at Arlington Arlington Texas USA; ^3^ Department of Orthopaedic Surgery University of California San Diego San Diego California USA

**Keywords:** artificial gravity, astronaut, bed rest, centrifugation, lower body negative pressure, microgravity, space travel, treadmill exercise

1

We read with interest the *Experimental Physiology* review article entitled ‘The search for the ultimate exercise countermeasure to preserve crew health and ensure mission success in long‐duration spaceflight’ by Fernandez‐Gonzalo et al. (2026). We similarly read more recent *Experimental Physiology* review and viewpoint articles entitled ‘Microgravity‐induced changes in skeletal muscle and possible countermeasures: What we can learn from bed rest and human space studies’ (Bosutti et al., 2026), and ‘From Earth to orbit: How to preserve muscle health in space and bed rest’ (Matsakas & Deane, 2026), respectively. While our letter focuses on the work by Fernandez‐Gonzalo et al. (2026), it also applies to the latter two papers in regards to microgravity‐induced muscle atrophy and artificial gravity (AG).

Fernandez‐Gonzalo and co‐authors appropriately describe several important features and goals of spaceflight countermeasure research and countermeasures themselves. Their article's premise is that extant countermeasure research fails to address these opportunities fully. We write to call attention to over three decades of research on lower body negative pressure (LBNP) treadmill exercise as a countermeasure that in fact addresses most of their concerns.

The authors correctly assert that load variability and control constitute key features of an ideal countermeasure. To that point, the ability to adjust and control *G_z_* (footward) force by adjusting LBNP was the seminal finding that launched investigation of LBNP exercise as a countermeasure (Hargens et al., 1991). Reducing air pressure around the lower body proportionally increases *G_z_* loading from 0 to >1 Earth body weight. Moreover, Moon and Mars gravities are simulated along this continuum.

Fernandez‐Gonzalo and co‐workers rightly emphasize the goal of an ‘exercise routine that can truly serve as a multi‐organ countermeasure.’ The discovery that LBNP generated a gravity‐like musculoskeletal stimulus complemented the well‐established cardiovascular effects of LBNP. Furthermore, increasing LBNP waist seal surface area halved the negative pressure needed to produce a given level of *G_z_* loading (Watenpaugh et al., 2000). This innovation thereby matched cardiovascular and musculoskeletal stimuli to accurately simulate gravity (Boda et al., 2000).

Since those early findings, bed rest simulations of microgravity demonstrated that LBNP treadmill exercise fully or partially protects anti‐gravity muscle mass and strength, bone density and metabolism, spinal structure and integrity, balance, sprinting ability, other aspects of neuromuscular control and performance, exercise capacity, orthostatic tolerance, and renal function. LBNP may also defend against spaceflight‐associated neuro‐ocular syndrome (SANS). The collective findings validate LBNP treadmill exercise as a form of AG.

The authors note that ‘The room available for both exercise devices and crew time is very limited during current space missions.’ Future missions will have similar limitations. These observations emphasize advantages of LBNP exercise over exercise during centrifugation as prospective countermeasures. The LBNP treadmill exercise protocol successfully employed in multiple bed rest studies is relatively time‐efficient at ≤45 min 4–6 days per week. A centrifuge exercise protocol capable of providing the same suite of benefits as LBNP exercise has not been devised regardless of duration. One complicating factor is that, unlike LBNP, short‐radius centrifugation often induces debilitating motion sickness. This and other issues raise safety and operational concerns about centrifugation.

The volume needed for a LBNP treadmill exercise device is roughly one‐fifth of that needed for the smallest possible short‐radius centrifuge exercise system. Torque from regularly starting and stopping a massive on‐board centrifuge demands much from a spacecraft's attitude control systems; LBNP imposes no such demand. The cost of a rotating spacecraft to produce long‐radius centrifugation is prohibitive compared to the cost of a treadmill in a LBNP chamber. In terms of research and engineering maturity, LBNP exercise AG is ready for in‐orbit testing, whereas centrifugation is far from this step.

Fernandez‐Gonzalo and co‐authors state that ‘Space agencies play an important role here and should encourage collaboration between research groups with different expertise by prioritizing multi‐organ projects.’ Interdisciplinary research of LBNP treadmill exercise has involved both multi‐institution and multi‐national teams of investigators funded by their respective space agencies. The most notable may be the WISE‐2005 project conducted at the Institute for Space Medicine and Physiology in Toulouse, France (MEDES; https://www.medes.fr/en/). That bedrest study included a component investigating exercise and dietary countermeasures, as suggested by Fernandez‐Gonzalo et al. (Lee et al., 2014).

The authors contend that enjoyment of exercise is an under‐recognized need. To that end, investigators proposed integration of virtual environments (VE) with LBNP treadmill exercise (Watenpaugh et al., 2000). Such technology could simulate, for example, running with others along an astronaut's favourite routes on Earth. Because adjustment of LBNP can simulate different gravity levels, integrated VE could also allow astronauts to explore and train for terrain features of their Moon, Mars or other extraterrestrial destination in advance of their arrival.

To account for innate variability between subjects and optimize countermeasure effectiveness, Fernandez‐Gonzalo and colleagues recommended individualization of exercise countermeasures according to user phenotype and responses. For all bed rest evaluations of LBNP treadmill exercise, interval protocol treadmill speeds were individualized to a given subject's baseline fitness level to elicit 40–80% of their peak oxygen consumption. Adjustment of LBNP allowed further individualization: in particularly fit subjects, LBNP could be increased to produce up to 1.2 body weight of *G_z_* loading.

Fernandez‐Gonzalo and co‐authors emphasize the importance of robust experimental design to minimize unwanted sources of variability. They recommend cross‐over designs in bed rest studies where each subject serves as their own control. Almost all bed rest studies of LBNP treadmill exercise to date used such cross‐over designs. One novel exception employed identical twins as subjects, with one serving as the sedentary control while the other exercised (Macias et al., 2007). Like cross‐over designs, this approach eliminated genetic variability between groups.

Journal limitations prevent citing references for all findings noted above, but source articles are easily found online. LBNP treadmill exercise may or may not be the ultimate exercise countermeasure, but a substantial body of peer‐reviewed literature suggests its inclusion in discussions of this topic. We thank the collective authors for their interesting and thought‐provoking articles, and for the opportunity they presented to share related information.

## AUTHOR CONTRIBUTIONS

Both authors contributed to the writing and editing of this letter. Both authors read and approved the final version of this letter, and both agree to be accountable for all aspects of the work. They will ensure that questions related to the accuracy or integrity of any part of the work are appropriately investigated and resolved. Both authors qualify for authorship, and all persons who qualify for authorship are included.

## CONFLICT OF INTEREST

The authors have nothing to report.

## FUNDING INFORMATION

This work was funded in part by Studio Videnda LLC.
